# Assessing the burden of outpatient urinary tract infections in the United States: analysis of nationwide ambulatory data (2016–2019)

**DOI:** 10.1017/ash.2025.10045

**Published:** 2025-06-30

**Authors:** Sonali D. Advani, Meghan E. Luck, Rose Chang, Mei Sheng Duh, Raj Desai, Megan Pinaire, Daisy Liu, Wendy Y. Cheng, Jeffrey J. Ellis

**Affiliations:** 1 Duke University School of Medicine, Durham, NC, USA; 2 GSK, Collegeville, PA, USA; 3 Analysis Group, Inc., Boston, MA, USA

## Abstract

We conducted an analysis of a nationwide survey of US physician offices between 2016 and 2019 and calculated annualized prevalence rates of urinary tract infections (UTIs). During the 3-year study period, UTI was the most common infection in US physician offices, accounting for approximately 10 million annualized encounters.

## Introduction

Urinary tract infection (UTI) is one of the most common infectious diseases encountered in the outpatient setting.^1^ Despite this burden, national measurement efforts focus primarily on device-related infections in hospitalized patients, requiring labor-intensive chart reviews and the application of complex definitions.^2^ Consequently, contemporary data on the prevalence of outpatient UTIs remain scarce.

Prior US studies have reported outpatient UTI burden through an economic lens. One study reported that complicated UTIs (cUTIs) accounted for 59% of the 30-day Medicare spending among adult beneficiaries.^3^ To fill current gaps in surveillance of infectious diseases, the US Department of Health and Human Services recommended leveraging clinician-assigned diagnosis during ambulatory care visits.^4^ Publicly available national databases like National Ambulatory Medical Care Survey (NAMCS) have been used to measure several outpatient conditions (eg sexually transmitted infections).^5,6^

The Centers for Disease Control and Prevention (CDC) identified outpatient UTI prevalence as a critical measurement gap in 2023.^7^ It is imperative to characterize the outpatient burden of UTIs to assess the need for and impact of stewardship interventions. This study aimed to (1) assess outpatient UTI burden, (2) evaluate the feasibility of using nationwide databases for UTI surveillance, and (3) suggest steps for improving future reporting.

## Methods

**Study Design:** We conducted a cross-sectional analysis of data from NAMCS between 2016 to 2019. NAMCS, a nationally representative survey of U.S. physician office visits, is conducted by the National Center for Health Statistics of the CDC.^5^ Data from 2017 and 2020 onwards were not available at the time of this study. Institutional review board approval was not required as this was a secondary analysis of publicly available data.

**Case Definitions:** UTI diagnosis required documentation of one of the following International Classification of Diseases (ICD), Tenth Revision, Clinical Modification (ICD-10-CM) diagnosis: acute cystitis (N30.0); other chronic cystitis (N30.2); other cystitis (N30.8); cystitis unspecified (N30.9); UTI, site not specified (N39.0); acute pyelonephritis (N10); non-obstructive reflux-associated chronic pyelonephritis (N11.0); chronic obstructive pyelonephritis (N11.1); pyonephrosis (N13.6). cUTI definitions were based on regulatory and professional society guidance at the time of the study (Supplement 1).^3^ UTIs that did not meet the cUTI definition were categorized as uncomplicated UTIs (uUTIs).

**Analysis:** We included UTI encounters in patients aged ≥15 years, calculated annualized prevalence rates, and stratified by uUTI and cUTI. Visit weights were applied to estimate national UTI prevalence. Specifically, we used the validated multiplicity estimator method^8^ to extrapolate visit-level data to patient-level estimates. This method reduces the contributions of patients with multiple encounters in a given year by multiplying visit weights by the inverse of the multiplicity factor, yielding a patient weight (Supplement 2). Patient weights were then applied to estimate the total number of patients with UTI, uUTI, and cUTI for each year. Prevalence was calculated per 100,000 people using U.S. Census Bureau data as the denominator. Statistical analyses were performed using SAS Enterprise Guide, Version 7 (SAS Institute, Cary, NC).

## Results

During the 3-year study period, NAMCS recorded 31,368 unweighted encounters, corresponding to 926,865,040 annualized weighted encounters. Of these, 372 were unweighted UTI encounters, equating to 9,799,054 (95% CI: 8,683,188–10,914,919) annualized weighted UTI encounters. (Supplement 3). The highest number of weighted UTI encounters was 12,208,220 (95% CI: 9,866,810–14,549,631) in 2019, and lowest in 2018 with 7,461,052 (95% CI: 6,457,715–8,464,389) encounters.

**Prevalence of UTIs:** Across the 3-year study period, the annualized prevalence of UTIs was 1,511 (95% CI: 1,234–1,787) per 100,000 persons. uUTI and cUTI prevalence rates were 851 (95% CI: 685–1,017) and 659 (95% CI: 495–824) per 100,000 persons respectively. The highest UTI prevalence was observed in patients aged 25–44 years (4,978 [95% CI: 4,640–5,316] per 100,000), followed by those aged ≥75 years (1,870 [95% CI: 1,281–2,460] per 100,000). UTIs were more prevalent in female patients (1,803 [95% CI: 1,486–2,120] per 100,000) than in males (966 [95% CI: 637–1,296] per 100,000). Among females, uUTI and cUTI prevalence rates were 1,473 (95% CI: 1,185–1,761) and 330 (95% CI: 231–428) per 100,000, respectively. All male UTIs were classified as cUTIs per existing guidelines, with most unweighted female cUTIs defined as such due to urologic abnormalities (92.5%).

**Characteristics of UTI encounters:** Among 9,799,054 annualized weighted UTI encounters, 4,133,236 (42.2%) were classified as cUTIs (Table 1). Over 55% of all weighted UTI encounters were in adults aged ≥65 years, 71.0% were in female patients, and 75.8% in White patients (Table 2). Other characteristics, including race, ethnicity, insurance coverage, morbidities, laboratory testing, and underlying diagnosis, are shown in Table 2. Most importantly, the diagnosis of “UTI, site not specified” was the most common infection-related diagnosis at physician office visits (in all patients and female patients). The greatest proportion of weighted UTI encounters was due to a visit for a new problem with less than three months onset (40.0%), followed by a routine visit for a chronic problem (24.6%), and a visit for a flare-up of a chronic problem (20.4%).

**Table 1.** Annual stratified encounters and prevalence of urinary tract infections (UTI)

	Total number of unweighted encounters	Total number of weighted encounters^*^ (95% CI)	Number of patients (95% CI)	Prevalence, per 100,000 people^†^ (95% CI)
2016				
UTI	187	9,727,889 (7,397,930–12,057,849)	3,734,433 (2,935,975–4,532,891)	1,448 (1,138–1,758)
uUTI	71	4,358,719 (3,733,875–4,983,563)	1,954,855 (1,452,858–2,456,853)	758 (563–953)
cUTI	116	5,369,170 (3,387,709–7,350,631)	1,779,578 (1,184,142–2,375,015)	690 (459–921)
2018				
UTI	102	7,461,052 (6,457,715–8,464,389)	3,327,903 (1,719,157–4,936,649)	1,269 (656–1,883)
uUTI	47	4,984,511 (4,101,549–5,867,473)	1,829,176 (1,119,966–2,538,386)	698 (427–968)
cUTI	55	2,476,541 (1,812,905–3,140,178)	1,498,728 (918,171–2,079,284)	572 (350–793)
2019				
UTI	83	12,208,220 (9,866,810–14,549,631)	4,775,429 (3,440,464–6,110,393)	1,812 (1,305–2,318)
uUTI	44	7,654,224 (7,096,515–8,211,934)	2,886,054 (1,811,947–3,960,162)	1,095 (687–1,502)
cUTI	39	4,553,996 (2,275,595–6,832,397)	1,889,375 (730,693–3,048,057)	717 (277–1,156)
Combined 3 yr (2016, 2018, 2019)^‡^				
UTI	372	9,799,054 (8,683,188–10,914,919)	3,945,922 (3,233,695–4,668,148)	1,511 (1,234–1,787)
uUTI	162	5,665,818 (5,279,694–6,051,942)	2,223,362 (1,788,841–2,657,882)	851 (685–1,017)
cUTI	210	4,133,236 (3,186,737–5,079,735)	1,722,560 (1,292,706–2,152,414)	659 (495–824)

CI, Confidence Interval; cUTI, complicated urinary tract infection; UTI, urinary tract infection; uUTI, uncomplicated urinary tract infection.

* Total number of encounters was calculated using sample weights, with each patient encounter weight accounting for selection probabilities, physician non-response, and other adjustments to reflect the universe of office-based patient visits in the US.

† The estimated numbers of prevalent UTI, uUTI, and cUTI cases per 100,000 people were calculated by dividing the number of patients with UTI, uUTI, and cUTI for each year by the total US population ≥15 years of age in the same year and multiplying by 100,000.

‡ Calculated as an average annualized estimate by dividing sampling weights by three (the total number of years of data).

**Table 2.** Demographics and clinical characteristics of stratified UTI encounters

	Overall			Female uUTI			Female cUTI			Male cUTI		
	Number of UTI encounters (unweighted) N (%)	Number of UTI encounters (annualized weighted) N (%)^*^^†^	95% CI	Number of UTI encounters (unweighted) N (%)	Number of UTI encounters (annualized weighted) ^*^^†^	95% CI	Number of UTI encounters (unweighted) N (%)	Number of UTI encounters (annualized weighted) N (%)^*^^†^	95% CI	Number of UTI encounters (unweighted) N (%)	Number of UTI encounters (annualized weighted) ^*^^†^	95% CI
Total	372	9,799,054	(8,683,188–10,914,919)	162	5,665,818	(5,279,694– 6,051,942)	80	1,291,941	(814,884– 1,768,998)	130	2,841,295	(2,075,149– 3,607,440)
Age												
15–44 yr	54 (14.5)	1,834,264 (18.7)	(1,238,406, 2,430,121)	33 (20.4)	1,233,044 (21.8)	(745,839, 1,720,250)	10 (12.5)	–	–	11 (8.5)	–	–
45–64 yr	96 (25.8)	2,515,740 (25.7)	(1,604,381, 3,427,098)	38 (23.5)	930,731 (16.4)	(516,446, 1,345,015)	22 (27.5)	–	–	36 (27.7)	1,291,221 (45.4)	(520,300, 2,062,141)
65–74 yr	91 (24.5)	2,689,123 (27.4)	(2,007,790, 3,370,456)	39 (24.1)	1,876,219 (33.1)	(1,337,132, 2,415,307)	18 (22.5)	–	–	34 (26.2)	457,146 (16.1)	(224,414, 689,878)
75+ yr	131 (35.2)	2,759,928 (28.2)	(2,216,509, 3,303,347)	52 (32.1)	1,625,824 (28.7)	(1,236,520, 2,015,127)	30 (37.5)	446,588 (34.6)	(266,520, 626,656)	49 (37.7)	687,516 (24.2)	(438,023, 937,009)
Sex												
Male	130 (34.9)	2,841,295 (29.0)	(1,892,694, 3,789,895)	0 (0.0)	–	–	0 (0.0)	–	–	130 (100.0)	2,841,295 (100.0)	(2,075,149, 3,607,440)
Female	242 (65.1)	6,957,759 (71.0)	(6,160,075, 7,755,443)	162 (100.0)	5,665,818 (100.0)	(5,279,694, 6,051,942)	80 (100.0)	1,291,941 (100.0)	(814,884, 1,768,998)	0 (0.0)	–	–
Race												
White	252 (67.7)	7,430,285 (75.8)	(6,402,448, 8,458,122)	115 (71.0)	4,616,364 (81.5)	(3,999,097, 5,233,631)	60 (75.0)	960,043 (74.3)	(609,223, 1,310,862)	77 (59.2)	1,853,878 (65.2)	(1,108,632, 2,599,124)
Black/African American	28 (7.5)	–	–	5 (3.1)	–	–	2 (2.5)	–	–	21 (16.2)	–	–
Other^†^	10 (2.7)	–	–	7 (4.3)	–	–	1 (1.3)	–	–	2 (1.5)	–	–
Unknown	82 (22.0)	1,487,469 (15.2)	(843,984, 2,130,954)	35 (21.6)	710,072 (12.5)	(382,914, 1,037,229)	17 (21.3)	–	–	30 (23.1)	536,893 (18.9)	(230,039, 843,748)
Ethnicity												
Hispanic or Latino	52 (14.0)	1,506,283 (15.4)	(707,298, 2,305,268)	19 (11.7)	–	–	17 (21.3)	–	–	16 (12.3)	–	–
Not Hispanic or Latino	254 (68.3)	6,915,435 (70.6)	(5,873,590, 7,957,280)	109 (67.3)	3,980,731 (70.3)	(3,383,824, 4,577,638)	48 (60.0)	710,811 (55.0)	(571,867, 849,754)	97 (74.6)	2,223,893 (78.3)	(1,470,393, 2,977,394)
Unknown	66 (17.7)	1,377,336 (14.1)	(854,153, 1,900,519)	34 (21.0)	997,617 (17.6)	(513,229, 1,482,004)	15 (18.8)	–	–	17 (13.1)	–	–
Insurance coverage												
Medicare	188 (50.5)	4,488,694 (45.8)	(3,706,961, 5,270,428)	79 (48.8)	2,767,725 (48.8)	(2,184,781, 3,350,669)	44 (55.0)	756,116 (58.5)	(369,701, 1,142,530)	65 (50.0)	964,854 (34.0)	(722,578, 1,207,129)
Private	131 (35.2)	4,072,622 (41.6)	(2,984,972, 5,160,273)	60 (37.0)	2,358,916 (41.6)	(1,770,084, 2,947,747)	22 (27.5)	–	–	49 (37.7)	1,458,075 (51.)	(671,884, 2,244,267)
Medicaid, CHIP, or other state–based program	32 (8.6)	636,774 (6.5)	(276,067, 997,481)	11 (6.8)	–	–	10 (12.5)	–	–	11 (8.5)	–	–
Other	4 (1.1)	–	–	3 (1.9)	–	–	0 (0.0)	–	–	1 (0.8)	–	–
Unknown	17 (4.6)	–	–	9 (5.6)	–	–	4 (5.0)	–	–	4 (3.1)	–	–
Comorbidities												
Diabetes (type 1 or type 2)	50 (13.4)	1,807,927 (18.5)	(1,247,353, 2,368,501)	20 (12.3)	–	–	11 (13.8)	–	–	19 (14.6)	–	–
Diabetes (unspecified)	24 (6.5)	–	–	7 (4.3)	–	–	4 (5.0)	–	–	13 (10.0)	–	–
Hypertension	171 (46.0)	4,566,828 (46.6)	(3,829,216, 5,304,440)	61 (37.7)	2,462,211 (43.5)	(1,837,024, 3,087,397)	42 (52.5)	737,149 (57.1)	(425,976, 1,048,322)	68 (52.3)	1,367,468 (48.1)	(1,095,298, 1,639,638)
Other chronic cystitis	36 (9.7)	610,136 (6.2)	(241,018, 979,254)	0 (0.0)	–	–	31 (38.8)	468,646 (36.3)	(113,208, 824,085)	5 (3.8)	–	–
Obesity	26 (7.0)	–	–	14 (8.6)	–	–	5 (6.3)	–	–	7 (5.4)	–	–
Diagnosis codes associated with uUTI^‡^												
UTI (site not specified)	264 (71.0)	7,070,747 (72.2)	(5,942,456, 8,199,038)	122 (75.3)	4,308,319 (76.0)	(3,561,636, 5,055,002)	43 (53.8)	777,823 (60.2)	(472,021, 1,083,625)	99 (76.2)	1,984,605 (69.8)	(1,222,797, 2,746,413)
Cystitis	128 (34.4)	2,925,286 (29.9)	(1,996,344, 3,854,227)	44 (27.2)	1,384,348 (24.4)	(815,240, 1,953,455)	51 (63.8)	671,438 (52.0)	(337,462, 1,005,414)	33 (25.4)	869,500 (30.6)	(619,753, 1,119,248)
Acute cystitis	49 (13.2)	1,440,863 (14.7)	(866,659, 2,015,067)	25 (15.4)	–	–	6 (7.5)	–	–	18 (13.8)	–	–
Other chronic cystitis	36 (9.7)	610,136 (6.2)	(241,018, 979,254)	0 (0.0)	–	–	31 (38.8)	468,646 (36.3)	(113,208, 824,085)	5 (3.8)	–	–
Cystitis (unspecified)	29 (7.8)	–	–	19 (11.7)	–	–	1 (1.3)	–	–	9 (6.9)	–	–
Other cystitis	8 (2.2)	–	–	0 (0.0)	–	–	7 (8.8)	–	–	1 (0.8)	–	–
Reasons for classifying as cUTI												
Urologic abnormalities	111 (29.8)	1,595,763 (16.3)	(1,100,221, 2,091,305)	0 (0.0)	–	–	74 (92.5)	1,095,208 (84.8)	(688,545, 1,501,871)	37 (28.5)	500,555 (17.6)	(305,398, 695,711)
Visit characteristics: major reason for visit												
New problem (less than 3 moonset)	130 (34.9)	3,920,745 (40.0)	(3,023,089, 4,818,401)	66 (40.7)	2,335,543 (41.2)	(1,738,147, 2,932,939)	24 (30.0)	–	–	40 (30.8)	1,210,575 (42.6)	(988,286, 695,711)
Chronic problem, routine	118 (31.7)	2,412,736 (24.6)	(1,850,287, 2,975,184)	43 (26.5)	1,305,049 (23.0)	(975,272, 1,634,826)	26 (32.5)	–	–	49 (37.7)	625,618 (22.0)	(464,745, 786,490)
Chronic problem, flare-up	71 (19.1)	1,996,265 (20.4)	(1,215,397, 2,777,132)	24 (14.8)	–	–	20 (25.0)	–	–	27 (20.8)	–	–
Visit characteristics: laboratory testing and imaging												
Urinalysis or urine dipstick	246 (66.1)	6,059,530 (61.8)	(4,884,120, 7,234,940)	113 (69.8)	3,632,848 (64.1)	(3,078,094, 4,187,603)	48 (60.0)	732,352 (56.7)	(368,033, 1,096,671)	85 (65.4)	1,694,330 (59.6)	(947,313, 2,441,346)
Culture, urine	147 (39.5)	3,182,528 (32.5)	(2,466,267, 3,898,789)	55 (34.0)	1,686,125 (29.8)	(1,337,066, 2,035,184)	32 (40.0)	471,761 (36.5)	(262,989, 680,534)	60 (46.2)	1,024,642 (36.1)	(665,615, 1,383,669)
Ultrasound	72 (19.4)	898,937 (9.2)	(472,228, 1,325,646)	18 (11.1)	–	–	20 (25.0)	–	–	34 (26.2)	338,165 (11.9)	(136,964, 539,366)
CT scan	20 (5.4)	–	–	6 (3.7)	–	–	5 (6.3)	–	–	9 (6.9)	–	–

CHIP, Children’s Health Insurance Program; CT, computed tomography; cUTI, complicated urinary tract infection; UTI, urinary tract infection; uUTI, uncomplicated urinary tract infection.

* Calculated as an average annualized estimate by dividing sampling weights by 3 (the total number of years of data).

† Characteristics that were observed in fewer than 30 unweighted encounters (denoted by “-”) did not meet the National Center for Health Statistics statistical reliability criteria (ie, estimates are not reliable) and therefore not presented.

‡ Categories are not mutually exclusive and therefore may not sum to 100%.

## Discussion

Our study confirms that UTIs are still the most common infection-related diagnosis in the U.S. ambulatory setting, accounting for approximately 10 million annualized weighted visits over three years. These numbers represent one of the most contemporary estimates of UTIs in office-based physician practices, demonstrating the feasibility of using publicly available databases for outpatient UTI surveillance.

Additionally, we saw differences in UTI burden across sexes, age groups, and race, indicating the need for targeted prevention and treatment strategies in specific populations. Over 55% of weighted UTI encounters occurred in older adults, who are at risk of antibiotic adverse events and an increased healthcare burden. Understanding these patterns is crucial for tailoring antimicrobial stewardship efforts to different demographic needs. Furthermore, the frequent use of the “UTI, site not specified” diagnosis code suggests potential misclassification due to challenges in distinguishing uncomplicated from complicated UTIs.

Our study has some limitations. While the multiplicity method reduces the contributions of patients who make multiple visits to a single provider within a 12-month period, patients who visit multiple outpatient providers throughout the year may still be counted more than once, potentially leading to an overestimation of the true prevalence of UTI. Estimates are limited to office-based physician practices and community health centers but could be expanded by using other databases, such as the Nationwide Inpatient Sample (NIS) and Nationwide Emergency Department Sample (NEDS). NAMCS diagnoses were not assigned using standardized case definitions and may need to be adapted as UTI definitions evolve. As a cross-sectional limited dataset, NAMCS lacks longitudinal patient-level data, culture results, and complete prescription data.^9^

Despite these limitations, nationwide survey data offer advantages for surveillance of outpatient infections like UTIs, where diagnostic tests or prescriptions may not always be required. Monitoring the burden and trends of these infections is crucial for stewardship efforts, specifically tracking trends for overdiagnosis or misclassification. To enhance data quality, linking national databases with electronic health records could develop automated reporting systems, replacing labor-intensive surveillance.^10^

In conclusion, this study presents a contemporary picture of the national burden of UTIs, uUTIs and cUTIs. The recently released NHCS interactive dashboard (https://www.cdc.gov/nchs/dhcs/prelim-hc-visits/index.htm) and public-use data files provide new opportunities for analyzing ambulatory UTI trends. Automated diagnosis information, especially when linked to electronic health record databases, represents a crucial tool for tracking outpatient infections, reviewing prescribing practices, and informing stewardship interventions.^10^

## Supporting information

10.1017/ash.2025.10045.sm001 Advani et al. supplementary material Advani et al. supplementary material

## Data Availability

Data used in this study are publicly available via the U.S. Centers for Disease Control and Prevention National Center for Health Statistics.

## Supplementary material

The supplementary material for this article can be found at https://doi.org/10.1017/ash.2025.10045
