# Supplementary material for: Assessing the burden of outpatient urinary tract infections in the United States: analysis of nationwide ambulatory data (2016–2019)
Source: Antimicrob Steward Healthc Epidemiol. 2025 Jun 30;5(1):e143. doi: 10.1017/ash.2025.10045 (PMC12224138; doi:10.1017/ash.2025.10045)
Supplement: Advani et al. supplementary material [file S2732494X25100454sup001.docx]

**SUPPLEMENTARY MATERIAL**

**Supplement 1:** Complicated UTI was defined by regulatory and professional society guidance at the time the study was conducted and in alignment with existing algorithms, comprising the presence of one or more of the following criteria (not mutually exclusive): infection and inflammatory reaction due to indwelling urinary catheter; acute pyelonephritis; non- obstructive reflux-associated chronic pyelonephritis; chronic obstructive pyelonephritis; tubulo-interstitial nephritis, not specified as acute or chronic; complicated/uncontrolled diabetes; immunosuppressed conditions; pregnancy; urologic abnormalities; fever; urosepsis; urological or nephrological procedures (eg catheter, surgery); treatment with intravenous antibiotics (identified using NAMCS-specific Medication Code 09951); male sex. See Table 1 for relevant diagnoses and procedures codes.

**Table 1.** ICD-10-CM and ICD-10-PCS codes for diagnoses and procedures indicative of complicated UTI

| **Condition/procedure** | **ICD-10 codes** |
| --- | --- |
| **ICD-10-CM diagnosis codes** | |
| Infection and inflammatory reaction due to indwelling urinary catheter | T83.51x |
| Acute pyelonephritis | N10 |
| Nonobstructive reflux-associated chronic pyelonephritis | N11.0 |
| Chronic obstructive pyelonephritis | N11.1 |
| Tubulo-interstitial nephritis, not specified as acute or chronic | N12 |
| Complicated/uncontrolled diabetes | E10.0x-E10.8, E11.0x-E11.8, E13.0x-E13.8 |
| Immunosuppressed conditions | B20, B97.35, D80, D81, D82, D83, D84, Z51.1, Z92.3, Z94 |
| Urologic abnormalities | C67.0, C67.1, C67.2, C67.3, C67.4, C67.5, C67.6, C67.7, C67.8, C67.9, N13.9, N39.9, N36.9, Z85.6, Z85.50, Z85.54, Z85.59, Z93.6, Z90.6, C79.11, C79.19, D41.9, D41.8, N82.1, N82.0, Q64.10, Q64.19, Q64.9, N20.0, N20.1, N20.2, N20.9, N21.0, N21.1, N21.8, N21.9, N22, N23, N13.70, N13.71, N13.5, N13.8, N13.4, R80.2, N30.10, N30.11, N30.20, N30.21, N30.30, N30.31, N30.80, N30.81, N30.40, N30.41, N32.0, N32.1, N32.2, N32.3, N32.9, N31.2, N31.8, N31.9, N36.44, N99.511, N99.512, N99.510, N99.518, A18.13, B37.42, B37.49, B89, B99.9, P00.1, N31.9, N11.0, N11.8, N10, N12, N16, N20.0, N20.1, N20.2, N20.9, I12.0, I12.9, I13.2, N18.3, N18.4, N18.5, N18.6, N18.9, N19, Z99.2, N28.1 |
| Fever | R50.9 |
| Urosepsis | A41.51 |
| Pregnancy | O00-O48, O60-O77, O80-O82, O85-O92, O98, O99, Z33, Z34, Z36, Z37, Z64.0 |
|  |  |
| **ICD-10-PCS codes** | |
| Pregnancy | 0DQP0ZZ, 0DQP3ZZ, 0DQP4ZZ, 0DQP7ZZ, 0DQP8ZZ, 0DQR0ZZ, 0DQR3ZZ, 0DQR4ZZ, 0JCB0ZZ, 0JCB3ZZ, 0Q820ZZ, 0Q823ZZ, 0Q824ZZ, 0Q830ZZ, 0Q833ZZ, 0Q834ZZ, 0TQB0ZZ, 0TQB3ZZ, 0TQB4ZZ, 0TQB7ZZ, 0TQB8ZZ, 0TQD0ZZ, 0TQD3ZZ, 0TQD4ZZ, 0TQD7ZZ, 0TQD8ZZ, 0TQDXZZ, 0U7C7ZZ, 0UCG0ZZ, 0UCG3ZZ, 0UCG4ZZ, 0UCM0ZZ, 0UJD7ZZ, 0UQ90ZZ, 0UQ93ZZ, 0UQ94ZZ, 0UQ97ZZ, 0UQ98ZZ, 0UQC0ZZ, 0UQC3ZZ, 0UQC4ZZ, 0UQC7ZZ, 0UQC8ZZ, 0UQG0ZZ, 0UQG3ZZ, 0UQG4ZZ, 0UQG7ZZ, 0UQG8ZZ, 0UQGXZZ, 0UQM0ZZ, 0UQMXZZ, 0US90ZZ, 0US94ZZ, 0US9XZZ, 0W3R0ZZ, 0W3R3ZZ, 0W3R4ZZ, 0W3R7ZZ, 0W3R8ZZ, 0W8NXZZ, 0WQNXZZ, 10900Z9, 10900ZA, 10900ZB, 10900ZC, 10900ZD, 10900ZU, 10903Z9, 10903ZA, 10903ZB, 10903ZC, 10903ZD, 10903ZU, 10904Z9, 10904ZA, 10904ZB, 10904ZC, 10904ZD, 10904ZU, 10907Z9, 10907ZA, 10907ZB, 10907ZC, 10907ZD, 10907ZU, 10908Z9, 10908ZA, 10908ZB, 10908ZC, 10908ZD, 10908ZU, 10A00ZZ, 10A03ZZ, 10A04ZZ, 10A07Z6, 10A07ZX, 10A07ZZ, 10A08ZZ, 10D00Z0, 10D00Z1, 10D00Z2, 10D07Z3, 10D07Z4, 10D07Z5, 10D07Z6, 10D07Z7, 10D07Z8, 10D17ZZ, 10D18ZZ, 10E0XZZ, 10H003Z, 10H00YZ, 10H073Z, 10H07YZ, 10J00ZZ, 10J03ZZ, 10J04ZZ, 10J07ZZ, 10J08ZZ, 10J0XZZ, 10J10ZZ, 10J13ZZ, 10J14ZZ, 10J17ZZ, 10J18ZZ, 10J1XZZ, 10J20ZZ, 10J23ZZ, 10J24ZZ, 10J27ZZ, 10J28ZZ, 10J2XZZ, 10P003Z, 10P00YZ, 10P073Z, 10P07YZ, 10Q00YE, 10Q00YF, 10Q00YG, 10Q00YH, 10Q00YJ, 10Q00YK, 10Q00YL, 10Q00YM, 10Q00YN, 10Q00YP, 10Q00YQ, 10Q00YR, 10Q00YS, 10Q00YT, 10Q00YV, 10Q00YY, 10Q00ZE, 10Q00ZF, 10Q00ZG, 10Q00ZH, 10Q00ZJ, 10Q00ZK, 10Q00ZL, 10Q00ZM, 10Q00ZN, 10Q00ZP, 10Q00ZQ, 10Q00ZR, 10Q00ZS, 10Q00ZT, 10Q00ZV, 10Q00ZY, 10Q03YE, 10Q03YF, 10Q03YG, 10Q03YH, 10Q03YJ, 10Q03YK, 10Q03YL, 10Q03YM, 10Q03YN, 10Q03YP, 10Q03YQ, 10Q03YR, 10Q03YS, 10Q03YT, 10Q03YV, 10Q03YY, 10Q03ZE, 10Q03ZF, 10Q03ZG, 10Q03ZH, 10Q03ZJ, 10Q03ZK, 10Q03ZL, 10Q03ZM, 10Q03ZN, 10Q03ZP, 10Q03ZQ, 10Q03ZR, 10Q03ZS, 10Q03ZT, 10Q03ZV, 10Q03ZY, 10Q04YE, 10Q04YF, 10Q04YG, 10Q04YH, 10Q04YJ, 10Q04YK, 10Q04YL, 10Q04YM, 10Q04YN, 10Q04YP, 10Q04YQ, 10Q04YR, 10Q04YS, 10Q04YT, 10Q04YV, 10Q04YY, 10Q04ZE, 10Q04ZF, 10Q04ZG, 10Q04ZH, 10Q04ZJ, 10Q04ZK, 10Q04ZL, 10Q04ZM, 10Q04ZN, 10Q04ZP, 10Q04ZQ, 10Q04ZR, 10Q04ZS, 10Q04ZT, 10Q04ZV, 10Q04ZY, 10Q07YE, 10Q07YF, 10Q07YG, 10Q07YH, 10Q07YJ, 10Q07YK, 10Q07YL, 10Q07YM, 10Q07YN, 10Q07YP, 10Q07YQ, 10Q07YR, 10Q07YS, 10Q07YT, 10Q07YV, 10Q07YY, 10Q07ZE, 10Q07ZF, 10Q07ZG, 10Q07ZH, 10Q07ZJ, 10Q07ZK, 10Q07ZL, 10Q07ZM, 10Q07ZN, 10Q07ZP, 10Q07ZQ, 10Q07ZR, 10Q07ZS, 10Q07ZT, 10Q07ZV, 10Q07ZY, 10Q08YE, 10Q08YF, 10Q08YG, 10Q08YH, 10Q08YJ, 10Q08YK, 10Q08YL, 10Q08YM, 10Q08YN, 10Q08YP, 10Q08YQ, 10Q08YR, 10Q08YS, 10Q08YT, 10Q08YV, 10Q08YY, 10Q08ZE, 10Q08ZF, 10Q08ZG, 10Q08ZH, 10Q08ZJ, 10Q08ZK, 10Q08ZL, 10Q08ZM, 10Q08ZN, 10Q08ZP, 10Q08ZQ, 10Q08ZR, 10Q08ZS, 10Q08ZT, 10Q08ZV, 10Q08ZY, 10S07ZZ, 10S0XZZ, 10T20ZZ, 10T23ZZ, 10T24ZZ, 10Y03ZE, 10Y03ZF, 10Y03ZG, 10Y03ZH, 10Y03ZJ, 10Y03ZK, 10Y03ZL, 10Y03ZM, 10Y03ZN, 10Y03ZP, 10Y03ZQ, 10Y03ZR, 10Y03ZS, 10Y03ZT, 10Y03ZV, 10Y03ZY, 10Y04ZE, 10Y04ZF, 10Y04ZG, 10Y04ZH, 10Y04ZJ, 10Y04ZK, 10Y04ZL, 10Y04ZM, 10Y04ZN, 10Y04ZP, 10Y04ZQ, 10Y04ZR, 10Y04ZS, 10Y04ZT, 10Y04ZV, 10Y04ZY, 10Y07ZE, 10Y07ZF, 10Y07ZG, 10Y07ZH, 10Y07ZJ, 10Y07ZK, 10Y07ZL, 10Y07ZM, 10Y07ZN, 10Y07ZP, 10Y07ZQ, 10Y07ZR, 10Y07ZS, 10Y07ZT, 10Y07ZV, 10Y07ZY, 2Y44X5Z, 30273H1, 30273J1, 30273K1, 30273L1, 30273M1, 30273N1, 30273P1, 30273Q1, 30273R1, 30273S1, 30273T1, 30273V1, 30273W1, 30277H1, 30277J1, 30277K1, 30277L1, 30277M1, 30277N1, 30277P1, 30277Q1, 30277R1, 30277S1, 30277T1, 30277V1, 30277W1, 3E0DXGC, 3E0E305, 3E0E329, 3E0E33Z, 3E0E36Z, 3E0E37Z, 3E0E3BZ, 3E0E3GC, 3E0E3HZ, 3E0E3KZ, 3E0E3NZ, 3E0E3SF, 3E0E3TZ, 3E0E705, 3E0E729, 3E0E73Z, 3E0E76Z, 3E0E77Z, 3E0E7BZ, 3E0E7GC, 3E0E7HZ, 3E0E7KZ, 3E0E7NZ, 3E0E7SF, 3E0E7TZ, 3E0E805, 3E0E829, 3E0E83Z, 3E0E86Z, 3E0E87Z, 3E0E8BZ, 3E0E8GC, 3E0E8HZ, 3E0E8KZ, 3E0E8NZ, 3E0E8SF, 3E0E8TZ, 3E0P3VZ, 3E0P7VZ , 4A0H74Z, 4A0H7CZ, 4A0H7FZ, 4A0H7HZ, 4A0H84Z, 4A0H8CZ, 4A0H8FZ, 4A0H8HZ, 4A0HX4Z, 4A0HXCZ, 4A0HXFZ, 4A0HXHZ, 4A0J72Z, 4A0J74Z, 4A0J7BZ, 4A0J82Z, 4A0J84Z, 4A0J8BZ, 4A0JX2Z, 4A0JX4Z, 4A0JXBZ, 4A1H74Z, 4A1H7CZ, 4A1H7FZ, 4A1H7HZ, 4A1H84Z, 4A1H8CZ, 4A1H8FZ, 4A1H8HZ, 4A1HX4Z, 4A1HXCZ, 4A1HXFZ, 4A1HXHZ, 4A1J72Z, 4A1J74Z, 4A1J7BZ, 4A1J82Z, 4A1J84Z, 4A1J8BZ, 4A1JX2Z, 4A1JX4Z, 4A1JXBZ |
| **Urological or nephrological procedures** |  |
| Urinary catheter | 0T2BX0Z, 0T2DX0Z, 0T7B0DZ, 0T7B3DZ, 0T7B4DZ, 0T7B7DZ, 0T7B8DZ, 0T7C0DZ, 0T7C3DZ, 0T7C4DZ, 0T7C7DZ, 0T7C8DZ, 0T7D0DZ, 0T7D3DZ, 0T7D4DZ, 0T7D7DZ, 0T7D8DZ, 0T9B00Z, 0T9B30Z, 0T9B40Z, 0T9B70Z, 0T9B80Z, 0T9C00Z, 0T9C30Z, 0T9C40Z, 0T9C70Z, 0T9C80Z, 0T9D00Z, 0T9D30Z, 0T9D40Z, 0T9D70Z, 0T9D80Z, 0T9DX0Z, 0TPB00Z, 0TPB0CZ, 0TPB0DZ, 0TPB30Z, 0TPB3CZ, 0TPB3DZ, 0TPB40Z, 0TPB4CZ, 0TPB4DZ, 0TPB70Z, 0TPB7CZ, 0TPB7DZ, 0TPB80Z, 0TPB8CZ, 0TPB8DZ, 0TPBX0Z, 0TPBXDZ, 0TPD00Z, 0TPD0CZ, 0TPD0DZ, 0TPD30Z, 0TPD3CZ, 0TPD3DZ, 0TPD40Z, 0TPD4CZ, 0TPD4DZ, 0TPD70Z, 0TPD7CZ, 0TPD7DZ, 0TPD80Z, 0TPD8CZ, 0TPD8DZ, 0TPDX0Z, 0TPDXDZ |
| Ureteral stent | 0T29X0Z, 0T9600Z, 0T9630Z, 0T9640Z, 0T9670Z, 0T9680Z, 0T9700Z, 0T9730Z, 0T9740Z, 0T9770Z, 0T9780Z, 0T9800Z, 0T9830Z, 0T9840Z, 0T9870Z, 0T9880Z, 0T760DZ, 0T763DZ, 0T764DZ, 0T767DZ, 0T768DZ, 0T770DZ, 0T773DZ, 0T774DZ, 0T777DZ, 0T778DZ, 0T780DZ, 0T783DZ, 0T784DZ, 0T787DZ, 0T788DZ, 0TP900Z, 0TP90CZ, 0TP90DZ, 0TP930Z, 0TP93CZ, 0TP93DZ, 0TP940Z, 0TP94CZ, 0TP94DZ, 0TP970Z, 0TP97CZ, 0TP97DZ, 0TP980Z, 0TP98CZ, 0TP98DZ, 0TP9X0Z, 0TP9XDZ |
| Ventricular shunt to urinary system | 0016077, 00160J7, 00160K7, 0016377, 00163J, 00163K7, 0016477, 00164J7, 00164K7 |
| Spinal subarachnoid-ureteral shunt | 001U077, 001U0J7, 001U0K7, 001U377, 001U3J7, 001U3K7 |
| Kidney surgeries | 0T900ZZ, 0T907ZZ, 0T908ZZ, 0T910ZZ, 0T917ZZ, 0T918ZZ, 0TC00ZZ, 0TC07Z, 0TC08ZZ, 0TC10ZZ, 0TC17ZZ, 0TC18ZZ, 0TH50YZ, 0TH53YZ, 0TH54YZ, 0TH57YZ, 0TH58YZ, 0TJ50ZZ, 0TP50YZ, 0TP53YZ, 0TP54YZ, 0TW50YZ, 0TW53Y, 0TW54YZ, 0TW57YZ, 0TW58YZ, 0T9000Z, 0T9070Z, 0T9080Z, 0T9100Z, 0T9170Z, 0T9180Z, 0T9040Z, 0T9140Z, 0TC03ZZ, 0TC04ZZ, 0TC13ZZ, 0TC14ZZ, 0T9040Z, 0T9140Z, 0TC03ZZ, 0TC04ZZ, 0TC13ZZ, 0TC14ZZ, 0TF33ZZ, 0TF34ZZ, 0TF43ZZ, 0TF44ZZ, 0T937ZZ, 0T938ZZ, 0T947ZZ, 0T948ZZ, 0TC30ZZ, 0TC40ZZ, 0T9370Z, 0T9380Z, 0T9470Z, 0T9480Z, 0TJ54ZZ, 0TJ58ZZ, 0T903ZX, 0T904ZX, 0T907ZX, 0T908ZX, 0T913ZX, 0T914ZX, 0T917ZX, 0T918ZX, 0T933ZX, 0T934ZX, 0T937ZX, 0T938ZX, 0T943ZX, 0T944ZX, 0T947ZX, 0T948ZX, 0TB03ZX, 0TB04ZX, 0TB07ZX, 0TB08ZX, 0TB13ZX, 0TB14ZX, 0TB17ZX, 0TB18ZX, 0TB33ZX, 0TB34ZX, 0TB37ZX, 0TB38ZX, 0TB43ZX, 0TB44ZX, 0TB47ZX, 0TB48ZX, 0T900ZX, 0T910ZX, 0T930ZX, 0T940ZX, 0TB00ZX, 0TB10ZX, 0TB30ZX, 0TB40ZX, 0TJ53ZZ, 0TJ57ZZ, 0T900ZZ, 0T903ZZ, 0T904ZZ, 0T907ZZ, 0T908ZZ, 0T910ZZ, 0T913ZZ, 0T914ZZ, 0T917ZZ, 0T918ZZ, 0T930ZZ, 0T933ZZ, 0T934ZZ, 0T937ZZ, 0T938ZZ, 0T940ZZ, 0T943ZZ, 0T944ZZ, 0T947ZZ, 0T948ZZ, 0T500ZZ, 0T510ZZ, 0T530ZZ, 0T540ZZ, 0T503ZZ, 0T513ZZ, 0T533ZZ, 0T543ZZ, 0T504ZZ, 0T514ZZ, 0T534ZZ, 0T544ZZ, 0T507ZZ, 0T508ZZ, 0T517ZZ, 0T518ZZ, 0T537ZZ, 0T538ZZ, 0T547ZZ, 0T548ZZ, 0T500ZZ, 0T503ZZ, 0T504ZZ, 0T507ZZ, 0T508ZZ, 0T510ZZ, 0T513ZZ, 0T514ZZ, 0T517ZZ, 0T518ZZ, 0T530ZZ, 0T533ZZ, 0T534ZZ, 0T537ZZ, 0T538ZZ, 0T540ZZ, 0T543ZZ, 0T544ZZ, 0T547ZZ, 0T548ZZ, 0TB00ZZ, 0TB03ZZ, 0TB04ZZ, 0TB07ZZ, 0TB08ZZ, 0TB10ZZ, 0TB13ZZ, 0TB14ZZ, 0TB17ZZ, 0TB18ZZ, 0TB30ZZ, 0TB33ZZ, 0TB34ZZ, 0TB37ZZ, 0TB38ZZ, 0TB40ZZ, 0TB43ZZ, 0TB44ZZ, 0TB47ZZ, 0TB48ZZ, 0TB00ZZ, 0TB03ZZ, 0TB04ZZ, 0TB07ZZ, 0TB08ZZ, 0TB10ZZ, 0TB13ZZ, 0TB14ZZ, 0TB17ZZ, 0TB18ZZ, 0TB30ZZ, 0TB33ZZ, 0TB34ZZ, 0TB37ZZ, 0TB38ZZ, 0TB40ZZ, 0TB43ZZ, 0TB44ZZ, 0TB47ZZ, 0TB48ZZ, 0TT30ZZ, 0TT34ZZ, 0TT37ZZ, 0TT38ZZ, 0TT40ZZ, 0TT44ZZ, 0TT47ZZ, 0TT48ZZ |
| Repair of cystocele and rectocele | 0JQC0ZZ, 0JQC3ZZ, 0JUC07Z, 0JUC0JZ, 0JUC0KZ, 0JUC37Z, 0JUC3JZ, 0JUC3KZ |
| Urinary manometry, cystometrogram, urethral sphincter electromyogram, uroflowmetry, urethral pressure profile, other nonoperative genitourinary system measurements | 4A0D7BZ, 4A0D8BZ, 4A1D7BZ, 4A1D8BZ, 4A0D73Z, 4A0D83Z, 4A1D73Z ,4A1D83Z, 4A0D75Z, 4A0D85Z, 4A1D75Z, 4A1D85Z, 4A0D7DZ, 4A0D8DZ, 4A1D7DZ, 4A1D8BZ, 4A1D8DZ, 4A0D7LZ, 4A0D8LZ, 4A1D7LZ, 4A1D8LZ |
| Therapeutic distention of bladder, irrigation of nephrostomy, pyelostomy, ureterostomy, ureteral catheter, cystostomy, indwelling urinary catheter, genitourinary instillation | 0T7B7ZZ, 0T7B8ZZ, 3C1ZX8Z, 3E1K38Z, 3C1ZX8Z, 3E1K38Z, 3C1ZX8Z, 3E1K38Z, 3C1ZX8Z, 0UH37YZ, 0UH38YZ, 0UH87YZ, 0UH88YZ, 0UHH7YZ, 0UHH8YZ, 0WHR73Z, 0WHR7YZ, 10A07ZX, 3E1K78Z, 3E1K88Z |
| Nonoperative removal of therapeutic device from urinary system (pyelostomy, nephrostomy, ureterostomy, ureteral catheter, cystostomy, etc. | 0TP5X0Z, 0TP9X0Z, 0TPBX0Z, 0TPDX0Z, 0TPDXDZ, 0TP57YZ, 0TP58YZ, 0TP97YZ, 0TP98YZ, 0TPB7YZ, 0TPB8YZ, 0TPD7YZ, 0TPD8YZ, 0WPRXYZ |
| Removal of intraluminal foreign body from uterus or urethra without incision, extracorporeal shockwave lithotripsy of the kidney, ureter/bladder | 0UC97ZZ, 0UC98ZZ, 0TCD7ZZ, 0TCD8ZZ, 0TCDXZZ, 0TF3XZZ, TF4XZZ, 0TF6XZZ, 0TF7XZZ, 0TFBXZZ, 0TFCXZZ, 0WFRXZZ |

**Supplement 2: Multiplicity Method calculation:**

To extrapolate visit-level data to patient-level estimates, the multiplicity estimator method was applied by accounting for the number of visits to the same provider in the past 12 months (i.e., the multiplicity factor). This method reduces the contributions of patients with multiple encounters in a given year by multiplying visit weights by the inverse of the multiplicity factor, yielding a patient weight.

For instance, if the visit weight was 25,661 and the patient had 7 total visits

- 6 visits in the past 12 months plus the current visit,
- the patient weight would be 25,661 × (1/7) = 3,665.

Patient weights were then applied to estimate the total number of patients with UTI, uUTI, and cUTI for each year.

**Supplement 2: Figure: Urinary Tract Infection Encounter Selection from Nationwide Ambulatory Medical Care Survey (NAMCS)**

**
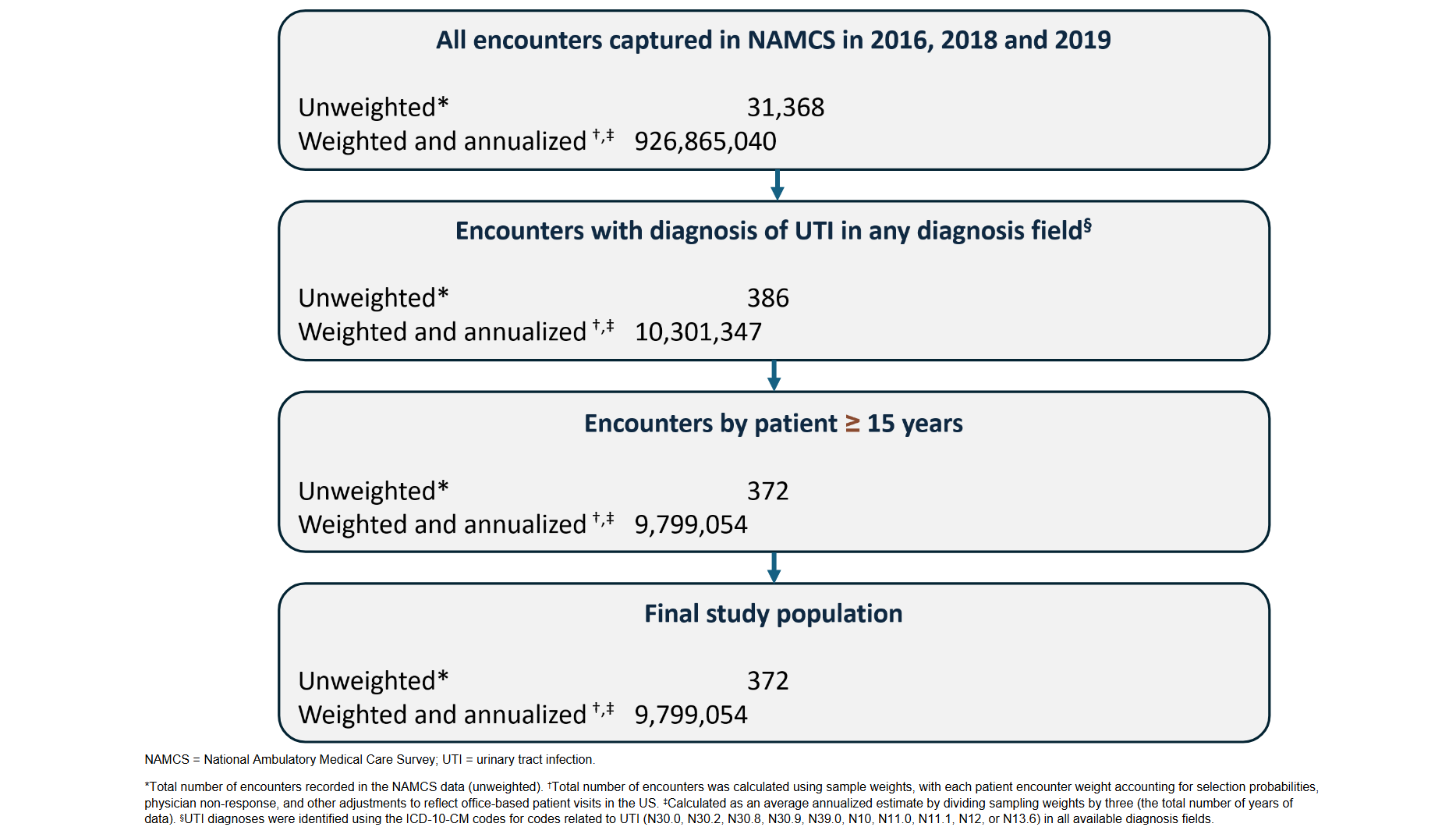
**
